# Case Report: Resolution of a cutaneous infection with methicillin-resistant *Staphylococcus pseudintermedius* with topical therapy in a cat with pemphigus foliaceus

**DOI:** 10.3389/fvets.2026.1822252

**Published:** 2026-04-17

**Authors:** Sin-Wook Park, Keon Kim, Woong-Bin Ro, Chang-Min Lee

**Affiliations:** 1 Department of Veterinary Internal Medicine, College of Veterinary Medicine and BK21 FOUR Program for Creative Veterinary Science Research Center, Chonnam National University, Gwangju, Republic of Korea; 2 SKY Animal Medical Center, Gwangju, Republic of Korea; 3 Department of Veterinary Internal Medicine, College of Veterinary Medicine, Jeju National University, Jeju, Republic of Korea

**Keywords:** feline autoimmune skin disease, immunosuppression, MRSP, multidrug resistance, one health, pyoderma

## Abstract

**Introduction:**

Pemphigus foliaceus (PF) is the most common autoimmune skin disease in cats. Long-term immunosuppressive therapy increases susceptibility to opportunistic infections. Methicillin-resistant *Staphylococcus pseudintermedius* (MRSP) is an emerging multidrug-resistant pathogen in companion animals.

**Case description:**

An 8-year-old spayed female Abyssinian cat with PF, managed with prednisolone and cyclosporine, developed localized superficial pyoderma caused by MRSP despite prior systemic antimicrobial therapy. Upon diagnosis of MRSP, systemic antibiotics were discontinued, and reinforcement of a strict regimen using 2% chlorhexidine and 10% povidone–iodine with topical gentamicin therapy led to complete resolution of the lesions. The patient achieved dermatologic remission without recurrence, allowing successful tapering and discontinuation of immunosuppressive therapy.

**Conclusion:**

This case suggests that localized MRSP skin infections in selected feline patients receiving immunosuppressive therapy may be successfully managed with topical-based management alone. Such an approach supports antimicrobial stewardship in veterinary medicine, highlighting that topical therapy may be considered a reasonable first step in selected patients with localized superficial skin infections before escalating to systemic antimicrobials.

## Introduction

Pemphigus foliaceus (PF) is the most common autoimmune skin disease in cats; this condition typically affects the face, ears, and feet and is managed with immunosuppressive therapy such as corticosteroids or cyclosporine (1, 2). Long-term immunosuppression increases susceptibility to secondary bacterial infections (3–5).

Methicillin-resistant *Staphylococcus pseudintermedius* (MRSP) is an emerging multidrug-resistant pathogen in companion animals, of which there is growing recognition in cats (6, 7). Beyond veterinary concerns, MRSP has zoonotic potential and relevance to the One Health framework (8, 9). Limited treatment options and reliance on critically important antimicrobials, such as vancomycin, highlight the need for antimicrobial stewardship and interest in topical or alternative strategies, especially for patients with localized infections (6, 10).

This report describes a cat with PF receiving immunosuppressive therapy that developed a localized skin infection caused by MRSP during treatment. The infection successfully resolved with adjunctive topical antimicrobial therapy without additional systemic agents. This case illustrates the potential role of topical management for selected localized resistant skin infections while limiting further systemic antimicrobial exposure.

## Case description

An 8-year-old, 4-kg, female spayed Abyssinian cat was referred to our hospital for worsening pruritic dermatologic lesions initially affecting the pinnae and progressively extending to other regions despite multiple courses of empirical medical treatment over a two-month period. According to the owner, all medications had been discontinued for approximately 1 week prior to presentation; before discontinuation, the cat had been receiving amoxicillin (15 mg/kg PO BID), enrofloxacin (5 mg/kg PO SID), prednisolone (0.5 mg/kg PO BID), and famotidine (0.5 mg/kg PO BID), and chlorpheniramine (0.2 mg/kg PO BID). Empirical ectoparasite control had also been attempted with applications of a topical imidacloprid/moxidectin formulation.

**Figure 1:**
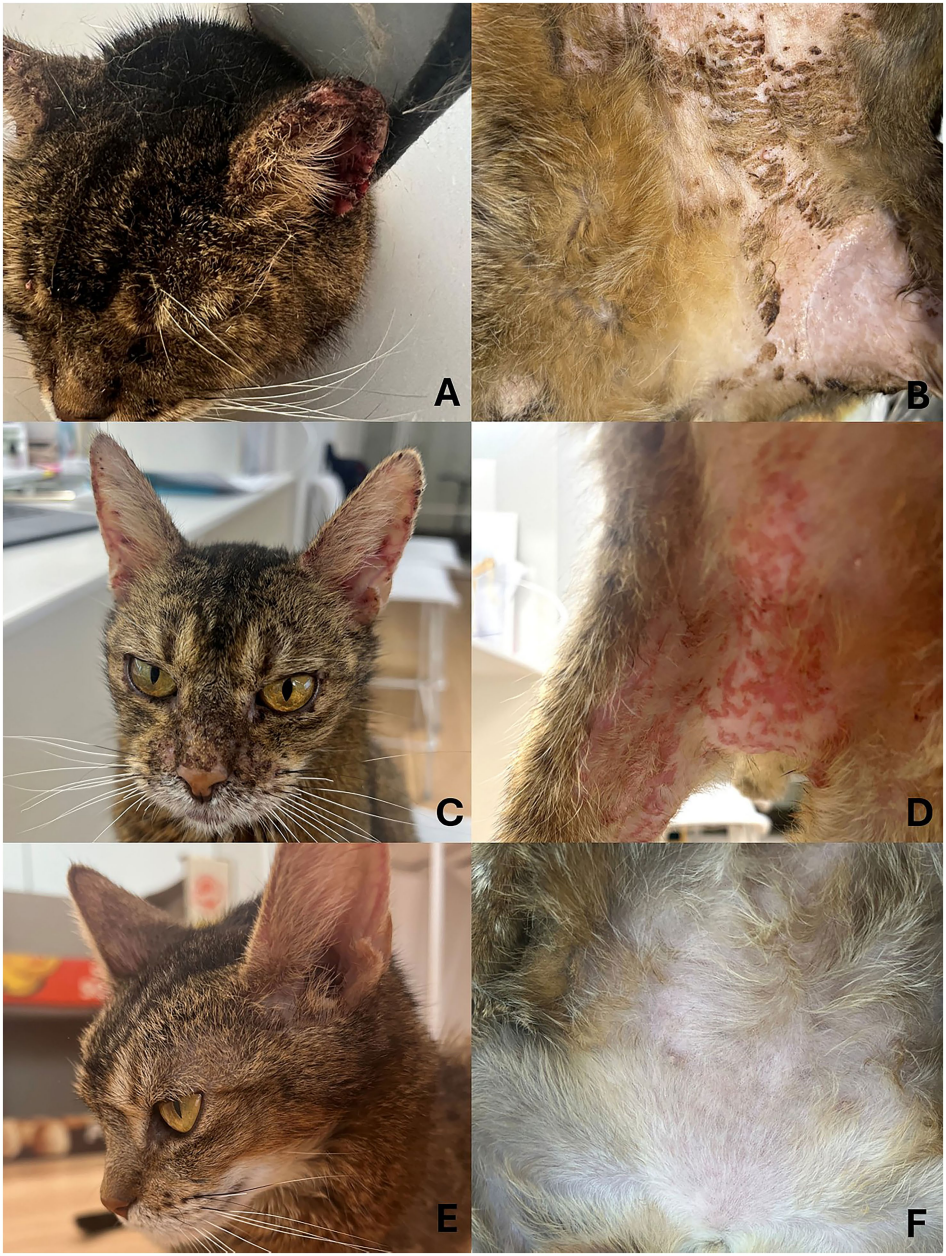
Sequential clinical images of a cat with pemphigus foliaceus (PF) before treatment, at the time of diagnosis of a methicillin-resistant *Staphylococcus pseudintermedius* (MRSP)-related skin infection during immunosuppressive therapy, and after resolution with topical management. **(A,B)** At presentation: marked crusting, erythema, excoriations, and alopecia affecting the pinnae **(A)** and multifocal papules and crusts in the inguinal region **(B)**. **(C,D)** During treatment at the time of diagnosis of MRSP: although the pinnal lesions showed overall improvement **(C)**, the erythema and papules in the right inguinal region were worse than those at presentation **(D**), from which MRSP was cultured. **(E,F)** After treatment completion: complete resolution of the lesions on the pinnae **(E)** and inguinal region with hair regrowth and no erythema or papules **(F)**.

At presentation, lesions were observed on the pinnae, ventral thorax, and inguinal region. The bilateral pinnae exhibited erythema, crusting, excoriation, and lichenification. The ventral body surface showed generalized alopecia with multifocal crusts and papules (Figure 1).

The initial diagnostic evaluation included a complete blood count, serum biochemistry, and endocrine testing. The cat was markedly anemic with a hematocrit of 13.3% (reference interval (RI): 30.3–52.3%) and had a leukocytosis with a white blood cell count of 20.29 × 103/μL (RI: 2.87–17.02 × 103/μL). Hypoalbuminemia (1.7 g/dL; RI: 2.3–3.9 g/dL) and a low total protein level (5.1 g/dL; RI: 5.7–8.9 g/dL) were noted. The patient’s total thyroxine was within the reference range at 1.9 μg/dL (RI: 0.8–4.7 μg/dL), and the serum amyloid A (SAA) level was markedly elevated at 100.45 mg/L (RI: 0–10 mg/L). A urinalysis was unremarkable. Abdominal radiographs and ultrasonography revealed no significant abnormalities. Feline immunodeficiency virus/feline leukemia virus antigen and antibody tests were negative. A blood smear evaluation revealed no significant morphological abnormalities to explain the anemia, and new methylene blue staining confirmed that it was a nonregenerative anemia. Polymerase chain reaction testing for hemotropic infection was negative. While chronic inflammation and immune-mediated processes were considered differentials for the anemia, further diagnostics including bone marrow evaluation and a direct Coombs’ test were declined by the owner.

**Figure 2:**
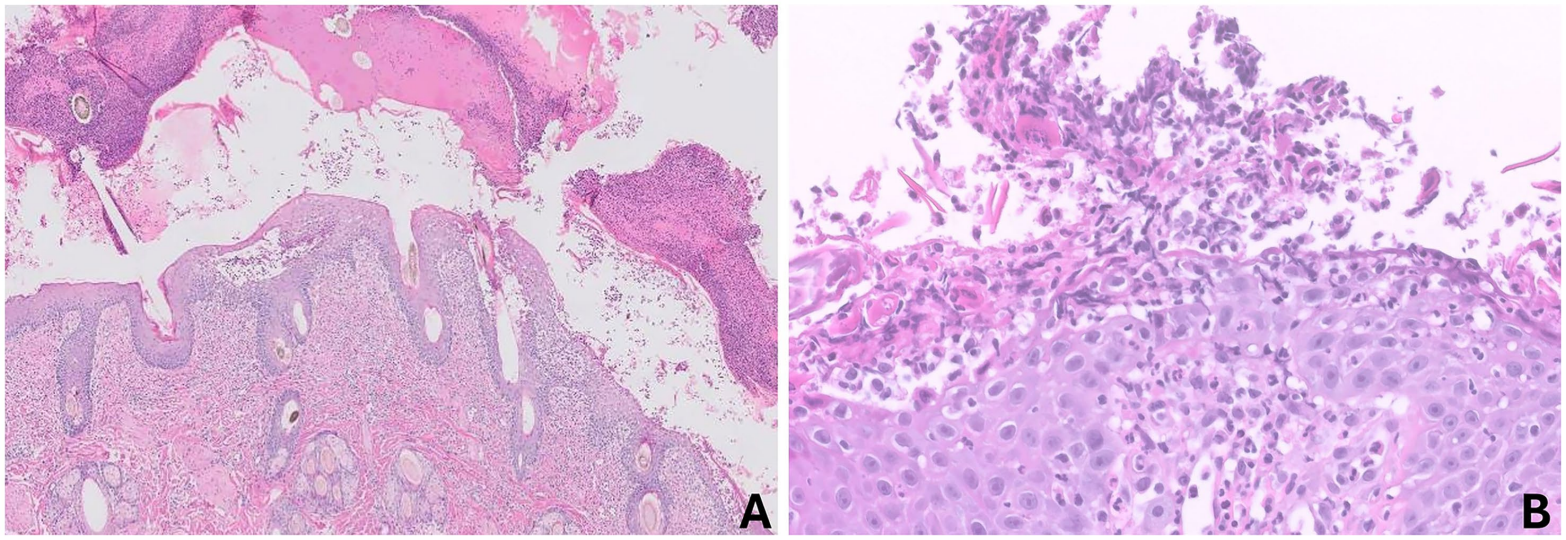
Histopathologic findings of a skin biopsy from the left inguinal region. **(A)** Low magnification (H&E, ×50) showing prominent surface crusting with marked epidermal hyperplasia and mixed dermal inflammation. **(B)** High magnification (H&E, ×400) revealing numerous acantholytic keratinocytes within pustules accompanied by neutrophils and colonies of cocci bacteria, consistent with a diagnosis of pemphigus foliaceus with a secondary bacterial infection.

Dermatologic diagnostics included impression cytology and a 4-mm punch biopsy from the left inguinal region. Cytology revealed numerous coccoid bacteria, neutrophils containing phagocytosed cocci, and clusters of degenerating/acantholytic keratinocytes. Bacterial isolates were identified by matrix-assisted laser desorption/ionization time-of-flight mass spectrometry (MALDI-TOF MS), and antimicrobial susceptibility testing was performed by the disk diffusion method. A swab sample for bacterial culture and antimicrobial susceptibility testing was collected from the left inguinal lesion after removal of overlying crust and sampling of the exposed lesional surface/papular contents. This yielded *Staphylococcus aureus*, which was susceptible to most tested antimicrobials (Table 1). Histopathologic analysis revealed neutrophilic and eosinophilic pustular dermatitis with acantholytic keratinocytes and surface crusts containing coccoid bacterial colonies (Figure 2). Periodic acid–Schiff staining was negative for dermatophytes. The clinicopathologic and histopathologic findings including characteristic lesion distribution and the presence of acantholytic keratinocytes were most consistent with PF. The bacterial growth was interpreted as a secondary infection associated with the underlying autoimmune skin disease.

**Table 1:** Bacterial isolates and antimicrobial susceptibility patterns before, during, and after treatment in a cat with PF and secondary infections.

Sampling time	Before treatment	During treatment	After treatment	Reference zone (mm)
Sampling site	Left inguinal	Right inguinal	Right inguinal	
Bacterial isolate	*Staphylococcus aureus*	*Staphylococcus pseudintermedius*	*Moraxella osloensis*	
Amikacin	S (18)	S (19)	S (28)	14–17
Vancomycin	S (18)	S (19)	S (22)	14–17
Amoxicillin/clavulanic acid	S (25)	R (6)	S (30)	19–20
Ampicillin	R (14)	R (6)	S (30)	28–29
Azithromycin	S (24)	R (6)	S (18)	13–18
Cefotaxime	S (32)	R (6)	S (25)	17–21
Cephalothin	S (28)	R (6)	S (26)	14–18
Clindamycin	S (28)	R (6)	S (27)	14–21
Cefovecin	S (33)	R (6)	S (30)	20–24
Doxycycline	S (26)	R (9)	S (23)	12–16
Enrofloxacin	S (25)	R (16)	S (33)	16–23
Imipenem	S (35)	R (6)	S (25)	13–16
Oxacillin	S (28)	R (6)	S (28)	17–18
Trimethoprim/Sulfamethoxazole	S (29)	R (6)	R (6)	10–16

Treatment was initiated with oral prednisolone (1.5 mg/kg PO BID), cyclosporine (25 mg/cat PO SID), amoxicillin-clavulanic acid (17.5 mg/kg PO BID), and famotidine (0.5 mg/kg PO BID). Topical therapy included Bedestar Cream (betamethasone dipropionate 640 μg/g, clotrimazole 10 mg/g, gentamicin sulfate 1 mg/g; Nelson Korea), which was applied to affected areas including the pinnae and ventral regions twice daily. In addition, 2% chlorhexidine solution and 10% povidone-iodine solution were used for antiseptic cleansing twice daily.

At a two-week recheck, the owner presented without the cat and reported that the skin lesions had improved during the first week of treatment, but pruritus, particularly around the ears, had recurred thereafter, accompanied by new lesions over the nasal planum. In response, the prednisolone dose was increased to 2 mg/kg PO BID and cyclosporine was increased to 25 mg/cat PO BID.

At the second follow-up visit 2 weeks after the treatment adjustment, partial clinical improvement was noted. The pinnae and ventral thorax showed marked reduction in erythema and crusting, whereas the right inguinal region exhibited worsening erythema and papules extending to the medial aspect of the right femoral region (Figure 1). The cat’s hematocrit had increased to 25.9%, with normalization of the white blood cell count and SAA. The serum albumin level also improved to 3.0 g/dL. As the anemia showed significant improvement without further clinical concerns, additional diagnostic testing for an underlying cause was not pursued. Cytology from the right inguinal lesion revealed degenerate neutrophils containing phagocytosed cocci. A second swab sample for bacterial culture and antimicrobial susceptibility testing was collected from an active right inguinal lesion after unroofing a papule and sampling the exposed lesional surface. Culture identified MRSP, which was resistant to all tested systemic antimicrobials except amikacin and vancomycin. As it was determined that the prescribed topical treatments had not been applied consistently, likely due to the extensive distribution of lesions and the patient’s poor compliance, the importance of strict adherence was reinforced. Intravenous vancomycin was considered; however, given the localized nature of the infection and absence of systemic illness, systemic therapy was deferred. The ongoing oral amoxicillin–clavulanic acid was discontinued, and immunosuppressive therapy was continued at the same dosages.

At the next recheck 2 weeks later, the inguinal and auricular lesions had markedly improved. However, the owner noted that the skin appeared thinner. To reduce the risk of corticosteroid-induced dermal atrophy, prednisolone was tapered to 1.5 mg/kg PO BID, and the topical steroid–antimicrobial cream was replaced with a gentamicin sulfate cream (1 mg/g; Shinpoong Pharm) for continued local antimicrobial coverage. Other medications were continued unchanged.

At the next recheck 2 weeks later, most dermatologic lesions had resolved. After clinical resolution, no active papules, pustules, or exudative lesions remained in the inguinal region. Therefore, the follow-up culture sample was obtained by gently swabbing only the superficial skin surface at the margin of the previously affected area, rather than from an active lesion. Follow-up bacterial culture of the right inguinal skin yielded *Moraxella osloensis*, which was interpreted as likely representing superficial recolonization or environmental contamination rather than ongoing infection. No further evidence of MRSP was found, suggesting successful resolution of the prior resistant infection. The prednisolone dose was tapered to 1 mg/kg BID, followed by a gradual tapering schedule leading to discontinuation. After successful withdrawal of prednisolone, cyclosporine was reduced to 25 mg PO SID and subsequently discontinued. Topical antiseptics were discontinued after approximately 1 month following complete resolution of dermatologic lesions. The cat received a total of 8 months of immunosuppressive therapy. At the time of discontinuation, all the clinical signs had resolved, and the hematologic parameters were within normal limits including a hematocrit of 35.2% and a consistently normal SAA concentration (Figure 1). During a telephone follow-up 1 year after treatment discontinuation, the owner reported that the cat remained clinically normal without recurrence of skin lesions.

## Discussion

An 8-year-old spayed female Abyssinian cat with PF was receiving immunosuppressive therapy when a secondary skin infection with MRSP was identified. The infection was managed successfully with topical therapy (antiseptics and a steroid–antimicrobial cream) while immunosuppressive medications were maintained, without the use of systemic antimicrobials active against MRSP. Complete dermatologic remission was achieved, permitting gradual tapering and eventual discontinuation of immunosuppressive medications, with no evidence of recurrence during follow-up. This case suggests that, in selected immunocompromised feline patients with localized multidrug-resistant skin infections, consistent topical therapy may provide effective infection control while supporting antimicrobial stewardship by reducing reliance on systemic antimicrobials.

PF is the most commonly reported autoimmune skin disease in cats and is characterized histologically by subcorneal pustule formation with numerous acantholytic keratinocytes and variable neutrophilic or eosinophilic infiltration (1). Lesions most frequently involve the face, ears, and distal limbs, although generalized distribution can occur (2). Long-term immunosuppressive therapy, typically with glucocorticoids alone or in combination with calcineurin inhibitors such as cyclosporine, remains the mainstay of treatment (11). While generally effective in controlling disease activity, such regimens increase susceptibility to opportunistic bacterial, fungal, or parasitic infections, which may complicate clinical management and adversely affect outcomes (12, 13). In the present case, PF lesions affecting the pinnae improved markedly with combined glucocorticoid and cyclosporine therapy; however, the inguinal region harboring MRSP exhibited worsening erythema and papules despite systemic immunosuppressive treatment. This emphasizes the importance of regular monitoring for secondary infections during immunosuppressive treatment, particularly when lesion distribution changes or localized deterioration is noted.

Immunosuppressive therapy predisposes feline patients to opportunistic infections by impairing immune defenses, altering cytokine signaling, and disrupting skin barrier integrity (13, 14). Such infections may arise from commensal organisms that become pathogenic under immunosuppressed conditions or from antimicrobial-resistant pathogens that are more difficult to eliminate (13). Secondary infections can delay recovery, complicate disease management, and necessitate adjustments to treatment protocols, thereby increasing the risk of disease relapse (15). In the present case, a multidrug-resistant bacterial skin infection was cultured from a localized lesion during active PF management. While culture results from a single site may not fully represent all affected areas, the detection of a resistant organism in any lesion warrants careful reassessment of ongoing therapy and consideration of targeted antimicrobial strategies.

Although *S. pseudintermedius* is more commonly recognized as a canine pathogen and appears to be less prevalent in cats, recent studies suggest that feline isolates may still carry a clinically meaningful resistance burden (12, 16, 17). In one epidemiologic study, multidrug resistance was detected in 50% of *S. pseudintermedius* isolates from healthy cats and 38.46% of isolates from sick cats, despite the overall low prevalence of this organism in the feline population (16). Other reports have likewise shown that, although *S. pseudintermedius* is isolated less frequently from cats than from dogs, feline isolates may still exhibit methicillin resistance or multidrug resistance (12, 18). Accordingly, although *S. pseudintermedius* appears to be less prevalent in cats, its isolation from active skin lesions may still be clinically meaningful, particularly when supported by cytologic evidence of infection.

In the present case, the discrepancy between the initial isolation of *S. aureus* and the subsequent isolation of MRSP should be interpreted cautiously. The two cultures were obtained from different inguinal lesions at different time points and therefore do not necessarily indicate a true shift in bacterial species within the same lesion. Possible explanations include lesion-to-lesion variation in bacterial populations, differences in organisms recovered from separate lesions, and the possible influence of interim antimicrobial exposure on subsequent culture results. Although the discrepant culture results should be acknowledged, the later isolation of MRSP from an active clinically worsening lesion, together with supportive cytologic findings, suggested that this organism was clinically relevant in the present case.

In cases of localized multidrug-resistant skin infections, topical therapy can achieve high local antimicrobial concentrations, reduce systemic drug exposure, and minimize the risk of adverse effects or further selection for resistance (19). Evidence from canine dermatology indicates that chlorhexidine-based antiseptic protocols can be effective in managing resistant staphylococcal infections when lesion distribution is limited and owner compliance is high (20, 21). In the present case, MRSP was confined to discrete cutaneous lesions, and systemic antimicrobial options were limited by the organism’s resistance profile and the potential for drug-associated adverse effects. Consistent application of topical antiseptics (chlorhexidine and povidone–iodine) in combination with a steroid–antimicrobial cream resulted in complete clinical resolution without recurrence, supporting the use of topical therapy alone as a feasible approach in selected immunocompromised feline patients with localized resistant infections (22). Immunosuppressive therapy was maintained because the infection appeared superficial, there was no evidence of systemic involvement, and topical treatment was anticipated to achieve sufficient bacterial clearance. This management strategy allowed continued control of the underlying autoimmune condition while avoiding systemic antimicrobial administration, consistent with principles of antimicrobial stewardship.

This case report has several limitations. The cause of the non-regenerative anemia at presentation was not definitively established; however, its improvement with immunosuppressive therapy suggests a relationship to severe systemic inflammation associated with PF or a possible immune-mediated process. The discrepant culture results should be interpreted in the context of the sampling conditions, as the cultures were obtained from different lesions at different time points, and the organisms recovered may therefore have been influenced by site-to-site variation and interim antimicrobial exposure. Owner compliance with follow-up visits was inconsistent, and in some instances, the patient was not presented for in-clinic evaluation. This limited our ability to assess lesion progression and response to treatment at all intended time points. In addition, the efficacy of individual components of the topical regimen could not be assessed, as they were used in combination.

This case highlights the clinical importance of antimicrobial stewardship in the management of resistant skin infections. It also demonstrates that, in selected feline patients receiving immunosuppressive therapy, clinical resolution of localized resistant skin infections may be achieved without additional systemic antimicrobial therapy through consistent topical management. These findings support consideration of topical therapy as a reasonable option in individualized treatment planning for selected cases.

## Data Availability

The original contributions presented in the study are included in the article/supplementary material, further inquiries can be directed to the corresponding authors.
